# A perspective on improving the teaching quality of aerospace cardiovascular medicine courses

**DOI:** 10.3389/fmed.2026.1860344

**Published:** 2026-08-18

**Authors:** Lin Cong, Meiqing Huang, Taihui Zhang, Wenlan Wang, Weitao Dang

**Affiliations:** School of Aerospace Medicine, The Fourth Military Medical University, Xi’an, Shaanxi, China

**Keywords:** aerospace cardiovascular medicine, medical education, project-based and interdisciplinary education, teaching case repository, teaching quality

## Abstract

With the rapid advancement of aerospace technology and continuous breakthroughs in the field of cardiovascular medicine, the teaching of aerospace cardiovascular medicine courses is facing new challenges. This narrative educational perspective analyzes and discusses the application of established medical education practices to aerospace cardiovascular medicine, including diverse teaching methods, technological tools, guidance and support systems, feedback and evaluation mechanisms, teaching case repositories, and project-based and interdisciplinary approaches. The aim is to contextualize these established practices through aerospace cardiovascular medicine-specific examples and to provide practical suggestions for educators in this specialized field.

## 1 Introduction

With continuing advances in aerospace technology and cardiovascular medicine, aerospace cardiovascular medicine education has become increasingly important (1). In this manuscript, aerospace cardiovascular medicine refers to an interdisciplinary field at the intersection of aerospace medicine and cardiovascular medicine that addresses cardiovascular physiological responses and adaptation, health risks, and preventive strategies associated with aviation and spaceflight environments, including hypobaric hypoxia, altered gravity, and radiation exposure (2, 3). Accordingly, this course not only covers fundamental medical knowledge but also addresses the effects of unique aerospace environments on the cardiovascular system, particularly cardiovascular physiological responses and adaptation (4). To cultivate professionals capable of working in this specialized field, improving the teaching quality of the course has become a critical task for universities (5).

Published reports, mainly from medical schools in the United States, indicate that aerospace medicine-related education is more commonly delivered through elective courses, short curricula, clerkships, student interest-group activities, and research experiences than through a standardized core course. These educational activities generally combine lectures on aviation and space physiology with case discussions, practical applications, career guidance, and supervised research or enrichment activities (6, 7). After completing medical school and the necessary postgraduate training, students interested in this field may pursue careers as aviation medical examiners, medical consultants to airlines or aviation authorities, aeromedical transport physicians, or researchers and educators. In the space medicine setting, students may also seek clinical and operational roles related to astronaut health, aerospace medicine research, or physician participation in human spaceflight programs (6, 8).

Despite these emerging educational pathways, the teaching of aerospace cardiovascular medicine still faces several challenges (9). On the one hand, the course content spans multiple domains with a complex knowledge system, requiring instructors to possess strong professional backgrounds and extensive teaching experience (10). On the other hand, due to rapid technological development in this field, teaching methods and tools must be continuously updated to meet new educational needs (11).

To address these challenges, this paper discusses how established medical education practices can be adapted to the specific physiological, clinical, and operational contexts of aerospace cardiovascular medicine. Its principal value lies in the discipline-specific examples and implementation suggestions provided for educators, rather than in proposing entirely new pedagogical methods. This article is a narrative educational perspective rather than a systematic review. The cited literature was used to support and contextualize the proposed teaching practices and aerospace cardiovascular medicine-specific examples. No prespecified systematic search or formal study-selection procedure was undertaken, and no statistical analysis or quantitative meta-analysis was performed. The literature was discussed narratively according to educational themes, without the use of a formal qualitative synthesis methodology or a study-quality or risk-of-bias assessment tool. Therefore, PRISMA reporting and protocol registration were not applicable.

## 2 Adoption of multiple teaching methods

Problem-based learning, interactive teaching, simulation-based training, and the flipped classroom are established approaches in medical education. Evidence from medical education research suggests that these approaches can support theoretical learning, practical performance, clinical reasoning, or learner engagement, although their effectiveness varies according to course design and implementation (12). In the present manuscript, their relevance is considered primarily through their application to aerospace cardiovascular medicine-specific physiological and operational scenarios. The discussion is organized into six interrelated themes: diverse teaching methods, technology-enhanced tools, personalized guidance and support, feedback and evaluation mechanisms, teaching case repositories, and project-based and interdisciplinary educational approaches.

**Figure 1:**
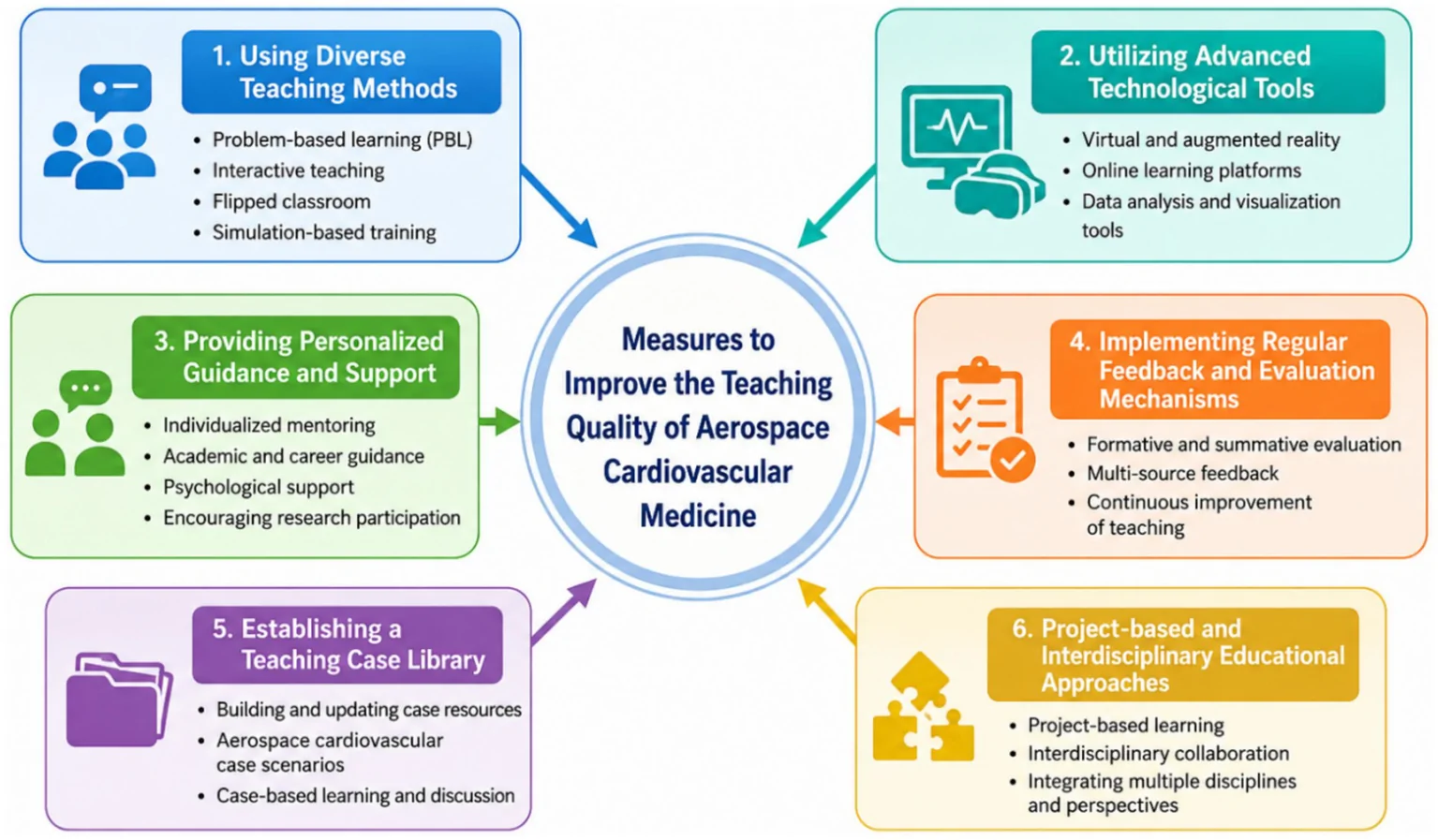
Author-developed conceptual framework for improving the teaching quality of aerospace cardiovascular medicine. The framework integrates the literature discussed in the corresponding sections with the authors’ analysis of discipline-specific educational needs and is not reproduced or adapted from a single external source.

Drawing on the medical education and aerospace medicine literature cited in the corresponding sections, together with the authors’ synthesis of the discipline-specific teaching needs of aerospace cardiovascular medicine, we developed the conceptual framework shown in Figure 1. This framework is an original author-generated synthesis and is not reproduced or adapted from any single published guideline or source.

### 2.1 Problem-based learning

Problem-based learning (PBL) is a student-centered, problem-oriented instructional method that places learners in real-world situations and encourages them to acquire knowledge and skills through independent inquiry and collaborative learning (13). In aerospace cardiovascular medicine courses, the particular value of PBL lies in organizing learning around uncommon cardiovascular problems and operational decisions that are rarely encountered in conventional medical curricula.

To effectively implement PBL, instructors must prepare thoroughly by designing appropriate problems and scenarios that guide students toward deeper thinking and hands-on practice (14). For example, a case scenario may present a situation in which a pilot conducting a cross-domain flight mission experiences abnormal heart rate and decreased blood pressure. Students, acting as flight surgeons, would need to assess the possible causes, determine the pilot’s fitness to continue the mission, and formulate an appropriate management plan.

PBL emphasizes critical thinking and collaboration. By presenting challenging problems, students apply theoretical knowledge to conduct analysis and develop solutions. This process stimulates critical thinking and allows learners to approach problems from multiple perspectives in search of optimal solutions. Collaboration is also encouraged, as solving complex issues often requires collective effort.

Given the complexity of medical and emergency management situations in aerospace settings, students must be able to translate theoretical knowledge learned in class into practical operational capability (15). Through PBL, learners apply cardiovascular medicine concepts in simulated aerospace medical scenarios, improving their understanding of real-world challenges.

### 2.2 Interactive teaching

Traditional one-way lectures alone may not fully meet the needs of contemporary medical education. In aerospace cardiovascular medicine courses, interactive teaching may increase learner engagement and support the development of critical thinking and problem-solving skills. Thus, instructors should design various interactive components—such as group discussions and role-playing—to encourage active participation (16).

To ensure that students better understand and apply what they learn, teaching content should be closely aligned with practical aerospace medical scenarios. For instance, real flight-related incidents can be introduced, allowing students to analyze and discuss cardiovascular changes and treatment strategies under special environmental conditions. The discipline-specific contribution of this approach is the use of flight-related cardiovascular events and role-based aeromedical decisions as the focus of discussion, rather than the interactive format itself.

Simulation-based training is also an important means of enhancing practical skills. In aerospace cardiovascular medicine courses, simulated flight missions and medical emergency drills should be incorporated (17). These activities allow students to experience cardiovascular issues that may occur during flight and help them develop emergency response competencies. Group discussions and role-playing can further stimulate creativity and broaden perspectives, while role-playing helps students understand responsibilities of various positions and improve communication skills.

### 2.3 Flipped classroom

The flipped classroom is an established blended-learning model in which learners review core content before class and use classroom time for discussion, application, and problem-solving (18). For a highly specialized and practice-oriented course such as aerospace cardiovascular medicine, the flipped classroom may be particularly useful because it allows classroom time to focus on the interpretation and management of complex aerospace cardiovascular scenarios.

First, it may enhance students’ interest and learning initiative. Rather than passively receiving information, students must prepare in advance, which promotes deeper comprehension of cardiovascular medicine concepts (19).

Second, the flipped classroom may support the development of practical skills. Students engage in discussions and hands-on activities during class, consolidating theoretical knowledge and developing operational and problem-solving skills. Instructors may pose open-ended questions—such as “How would you address tachycardia occurring during spaceflight?”—and ask students to propose solutions. Case materials may also be provided for diagnostic analysis.

To help students better understand aerospace physiology, simulated experiments or practical activities may also be arranged—for example, recording and analyzing ECG signals during simulated spaceflight conditions. Guest lectures from industry experts can further expose students to the latest research and applied practices.

The flipped classroom may allow instructors to place greater emphasis on independent learning and practical skill development while reserving classroom time for complex aerospace cardiovascular scenarios. This format may also help instructors identify learners’ needs and provide more targeted guidance.

## 3 Utilizing advanced technological tools

In addition to these established teaching methods, technology-enhanced tools can support the teaching of aerospace cardiovascular medicine. Tools such as virtual reality, online education platforms, and data analysis software can provide learning experiences related to complex or difficult-to-reproduce aerospace environments and physiological responses.

### 3.1 Virtual reality and augmented reality technologies

For students of aerospace medicine, understanding cardiovascular changes that occur under high-altitude and high-stress conditions is crucial. Virtual reality (VR) technology can create highly realistic flight environments in which visual and auditory stimuli simulate the sensory experiences encountered during flight (10). VR can reproduce various flight conditions and emergency scenarios, enabling students to observe and understand cardiovascular system responses during simulated flights (20). Through this immersive experience, students gain deeper insight into cardiovascular mechanisms and coping strategies in extreme environments.

VR can also be used to simulate emergency situations and clinical cases in aerospace medicine, such as cardiac arrest in astronauts. In a virtual environment, students can practice resuscitation and treatment procedures, improving their emergency response capabilities and practical skills (21). This form of simulated training enhances hands-on competence while reducing the risks and costs associated with real-life operations.

Augmented reality (AR) superimposes digital information on the physical environment and can support the visualization of spatial anatomical and physiological relationships. In aerospace cardiovascular medicine courses, AR can be used to visualize the structure and function of the cardiovascular system, allowing students to understand its components and mechanisms more intuitively (21).

For example, instructors can create three-dimensional cardiovascular models that students can view and interact with through AR devices (22). By rotating, zooming, and exploring these models, learners gain a comprehensive understanding of cardiovascular structure and characteristics. This interactive approach may support spatial understanding and learner engagement, although its effectiveness depends on the instructional design and learning task.

AR can also simulate surgical procedures by overlaying virtual surgical scenes and instruments onto real-world environments. Students can practice surgical techniques and emergency responses in a virtual context, strengthening their clinical and operational skills (23). Such simulation-based practice not only supports mastery of procedural skills but also prepares students for future real-world medical responsibilities.

**Figure 2:**
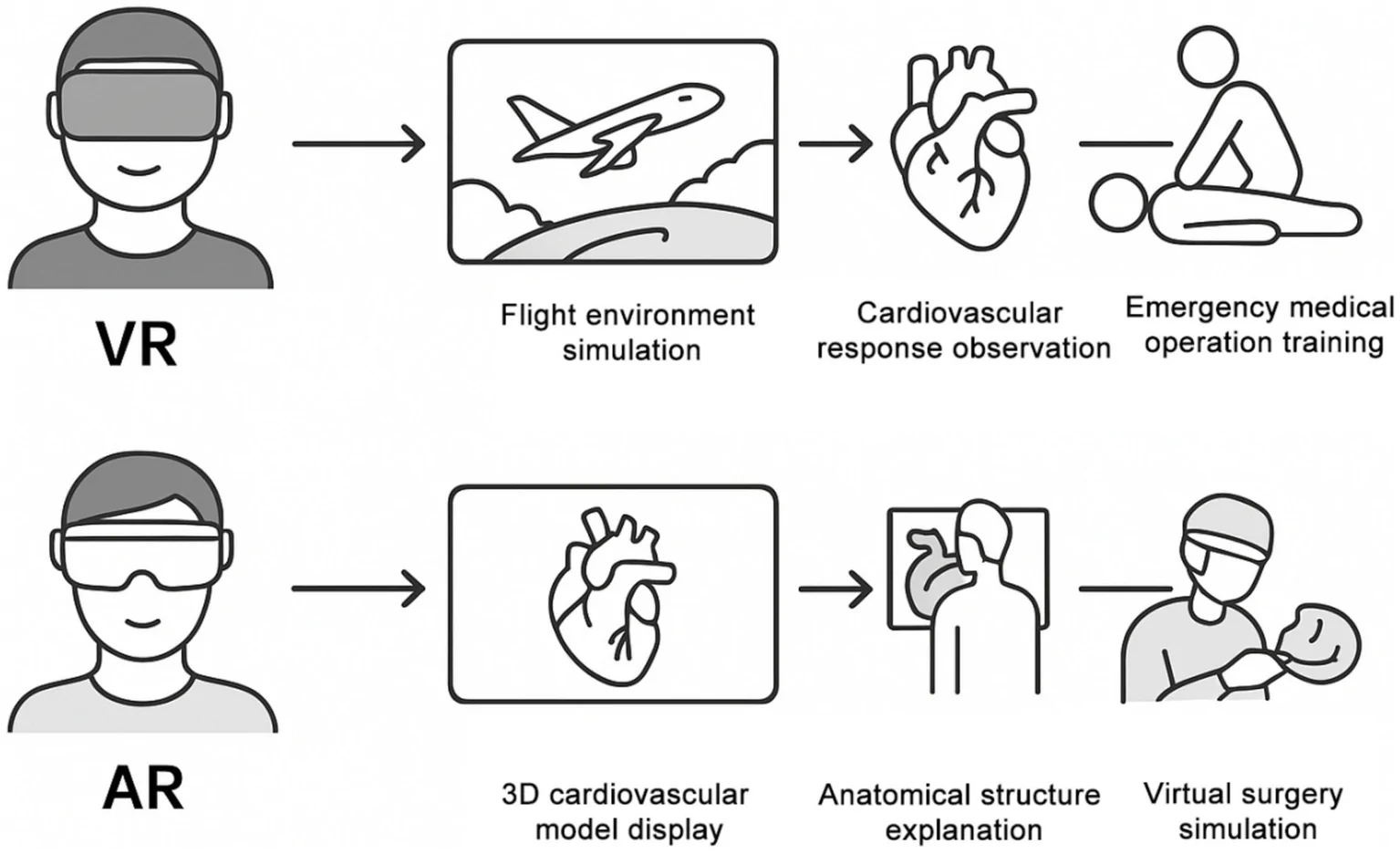
Schematic representation of VR/AR applications in aerospace cardiovascular medicine education.

An illustration of VR/AR applications in classroom teaching is presented in Figure 2.

### 3.2 Online education platforms

Online education platforms can integrate a wide range of resources related to aerospace cardiovascular medicine, such as instructional videos, expert lectures, and professional literature. These resources provide students with on-demand access to comprehensive knowledge about cardiovascular changes and challenges in aerospace environments.

Through real-time communication features offered by online platforms, instructors can conduct online Q&A sessions, discussions, and interactive activities with students. This can support timely problem-solving and learner engagement.

Furthermore, a specialized online community for aerospace cardiovascular medicine can be established, bringing together experts, instructors, and students. In this community, members can share experiences, exchange insights, and collectively improve teaching quality and professional competence (24).

Most online platforms also support mobile access, allowing students to study anytime and anywhere. This convenience improves learning efficiency and facilitates continuous knowledge updating. By effectively utilizing online education platforms, the teaching of aerospace cardiovascular medicine can achieve greater flexibility and efficiency, supporting distributed learning models.

### 3.3 Data analysis tools

The field of aerospace cardiovascular medicine involves diverse and complex datasets, including physiological data from pilots in high-altitude environments and disease monitoring data. To help students better understand and analyze such information, data-analysis environments such as Python and R may be introduced (25). These tools assist students in efficiently processing, cleaning, and organizing data.

By using data analysis software, students develop a data-driven mindset. Visualization tools like Tableau or Power BI can then help them observe trends and underlying patterns more intuitively, enhancing their understanding of key issues in the field (26). This data-oriented approach is valuable for future problem-solving and decision-making in professional practice.

The introduction of data analysis tasks can also increase classroom interactivity and student participation. Instructors may assign data analysis projects that require independent work, followed by presentations and discussion in class. In aerospace medicine, complex challenges—such as physiological reactions during high-altitude exposure or health risks associated with long-duration flights—can be explored through such assignments. This not only strengthens students’ analytical abilities but also helps them appreciate the real-world significance of data analysis.

## 4 Providing personalized guidance and support

Instructors engaged in aerospace cardiovascular medicine teaching should receive targeted and personalized guidance. Given differences in professional background, teaching experience, and instructional style, a dedicated mentoring team may be established to offer tailored support. This guidance may include course design, teaching methods, student engagement strategies, and research support (23), ensuring that each instructor receives comprehensive assistance and opportunities for growth.

Similarly, because students vary in their learning needs and abilities, personalized learning support should be provided. This may include customized study plans, resource recommendations, and practical opportunities that meet their individual learning requirements in aerospace cardiovascular medicine. Students should also be encouraged to raise questions and express concerns, with timely responses and guidance available.

To further enhance teaching quality, communication and collaboration between instructors and students should be strengthened. Regular seminars, academic activities, and discussion sessions can facilitate the exchange of ideas, helping both teachers and learners grow together.

Universities and departments should foster an open, inclusive, and innovative learning environment, encouraging experimentation and creativity. Student learning experiences and feedback should be emphasized, and teaching strategies adjusted accordingly to ensure continuous improvement in teaching quality.

## 5 Establishing regular feedback and evaluation mechanisms

To improve the teaching quality of aerospace cardiovascular medicine courses, it is essential to establish regular feedback and evaluation mechanisms. As the direct beneficiaries of teaching, students’ feedback is critical for quality enhancement. Regular collection of students’ evaluations—including feedback on teaching content, instructional methods, and instructor performance—should be conducted. Through questionnaires, individual interviews, and group discussions, students can be encouraged to express their views and experiences, allowing instructors to better understand their learning needs and expectations.

In addition to student feedback, peer review and expert consultation are also important for improving teaching quality. Departments may invite experts and scholars in related fields to conduct periodic evaluations of the aerospace cardiovascular medicine curriculum. Their professional insights and expertise can provide valuable suggestions regarding course design, teaching methods, and instructional effectiveness. Meanwhile, communication and discussion among peers can broaden instructional perspectives and foster innovation in teaching approaches.

Combining student evaluations with peer review can better identify areas for improvement in teaching strategies and methods. Before adopting feedback, instructors must analyze and assess the suggestions to distinguish truly valuable and constructive insights. First, the consistency of feedback should be considered—if multiple students or experts highlight the same issue or recommendation, it deserves focused attention. Second, the feasibility of the suggestions should be evaluated—whether they can be translated into actionable improvements. Finally, the scientific and pedagogical validity of the feedback should be reviewed—ensuring alignment with educational principles and teaching objectives.

Based on the evaluation results, instructors should acknowledge existing issues and actively implement improvements, adjusting teaching strategies and methods in a timely manner.

## 6 Establishing a teaching case repository

In the teaching of aerospace cardiovascular medicine, traditional instructional methods often emphasize theoretical knowledge while lacking practical case support. This results in a substantial gap between theory and practice, making it difficult for students to fully grasp and apply what they have learned. Therefore, establishing a teaching case repository that integrates real-world cases with theoretical instruction is essential for enhancing teaching quality (27).

Real cases in aerospace cardiovascular medicine often involve complex physiological, pathological, and environmental factors. Through case analysis and discussion, students can connect abstract theoretical knowledge with concrete real-world scenarios, enabling deeper understanding. This not only reinforces learning but also improves knowledge retention and application skills.

For example, a wide range of cardiovascular cases from aerospace settings can be collected—such as cardiovascular changes during astronaut missions and emergency medical responses. These cases can be organized, classified, and annotated to form a comprehensive case repository. Experts may be invited to analyze each case, identifying key issues and developing corresponding solutions. Students should also be encouraged to participate in case discussions to cultivate critical thinking and problem-solving abilities.

To ensure the quality and relevance of the case repository, continuous collection, review, and updating of cases are necessary. Instructors should receive appropriate training to master case-based teaching techniques, such as selecting suitable cases and guiding student case analysis. Students should also be encouraged to actively engage in discussions and provide insights that help further refine the case repository and teaching approaches.

## 7 Project-based and interdisciplinary educational approaches

With the advancement of technology and societal changes, educational methods are continuously evolving. Established approaches such as project-based learning and interdisciplinary teaching can be adapted to enhance students’ practical abilities, increase instructional interaction, and promote integrated disciplinary development in aerospace cardiovascular medicine. Therefore, instructors should adapt these established approaches to the characteristics and practical requirements of aerospace cardiovascular medicine and evaluate their effectiveness using clearly defined learning outcomes.

### 7.1 Project-based learning

Project-based learning aims to drive student development through hands-on project experiences. This method encourages active student participation and integrates theoretical knowledge with practical skills through teamwork and real-world problem-solving (28).

Students can engage in project tasks derived from their instructors’ research needs, such as developing rapid emergency intervention techniques for in-flight myocardial infarction or arrhythmia events. Each group may conduct literature reviews, design technical solutions, and discuss and optimize strategies.

Such project-based learning not only helps students apply theoretical concepts to practical scenarios but also enhances their adaptability, teamwork, and problem-solving abilities. Through close collaboration, students also deepen their understanding of aerospace cardiovascular medicine.

### 7.2 Interdisciplinary teaching

Interdisciplinary teaching integrates knowledge and skills from multiple fields into the learning process, helping students understand concepts more comprehensively and apply them more effectively. This approach fosters innovation, enhances overall competency, and supports the development of interdisciplinary thinking (29).

Because aerospace medicine intersects with numerous fields—including medicine, engineering, and physics—cardiovascular medicine within this context has unique disciplinary characteristics (30). Therefore, implementing interdisciplinary teaching is essential for improving the quality of aerospace cardiovascular medicine education.

Instructors may invite experts from various disciplines—such as aerospace engineering or biomedical engineering—to participate in teaching. Through interdisciplinary collaboration, students learn to approach problems from different perspectives, enabling them to better understand and apply their knowledge and skills in aerospace cardiovascular medicine. This approach may support interdisciplinary thinking and the development of professional competence.

## 8 Discussion

The six themes considered in this perspective indicate that improving aerospace cardiovascular medicine education is unlikely to depend on any single teaching method. Rather, the key requirement is to adapt and coordinate established educational practices according to the distinctive physiological, clinical, occupational, and operational problems encountered in aviation and spaceflight. This interpretation is consistent with broader medical education evidence suggesting that problem-based learning, simulation-based training, flipped classrooms, and other active-learning strategies may support knowledge acquisition, practical performance, clinical reasoning, and learner engagement, although their effectiveness varies with course design and implementation (12). It is also consistent with reports showing that aerospace medicine education is commonly delivered through electives, short curricula, student interest groups, and research experiences rather than through standardized core courses (6, 7, 25). These observations support the need for a coherent framework that connects general medical education practices with the specialized competencies required in aerospace cardiovascular medicine.

The significance of the proposed framework lies primarily in its discipline-specific integration. Learners must connect conventional cardiovascular knowledge with cardiovascular responses to hypobaric hypoxia and altered gravity, aeromedical fitness assessment, limited diagnostic resources, and mission-related decision-making. Simulation, VR/AR, and structured teaching cases may therefore be particularly useful for representing rare, high-risk, or difficult-to-reproduce scenarios. Nevertheless, technology-enhanced education should not be assumed to be inherently superior to conventional teaching. A recent systematic review and meta-analysis found that VR produced a moderate improvement in anatomy knowledge, whereas AR did not demonstrate a significant advantage, and substantial heterogeneity was observed across studies (31). This evidence suggests that technological tools should be selected according to learning objectives, scenario requirements, faculty capability, accessibility, and cost rather than technological novelty alone.

The interdisciplinary nature of aerospace cardiovascular medicine also supports the integration of cardiovascular medicine, aerospace physiology, biomedical engineering, data analysis, occupational health, and flight operations. A recent systematic review and meta-analysis of randomized trials found that interdisciplinary simulation-based education was beneficial for increasing interprofessional knowledge among healthcare learners (32). In the context of aerospace cardiovascular medicine, this supports the use of scenarios in which students jointly consider physiological adaptation, cardiovascular risk, engineering limitations, medical resources, and mission priorities. The educational value of such activities is likely to depend on careful scenario design, clearly assigned professional roles, structured facilitation, and post-simulation debriefing.

This perspective has several strengths. It integrates established medical education practices within a single aerospace cardiovascular medicine-specific framework and provides practical examples involving in-flight cardiovascular events, altered-gravity adaptation, aeromedical fitness assessment, emergency decision-making, and interdisciplinary mission requirements. However, several limitations should be acknowledged. This article was not based on a systematic literature search, formal study-selection procedure, or quality assessment; therefore, the cited literature may not represent all available evidence. In addition, many proposed applications remain conceptual and have not been evaluated specifically in aerospace cardiovascular medicine courses. Differences in faculty expertise, learner background, curriculum structure, technological resources, and institutional priorities may also affect feasibility and transferability. Accordingly, the proposed framework should be regarded as a practical educational perspective rather than a validated guideline or consensus standard.

For educational practice, institutions may begin by defining core aerospace cardiovascular competencies, selecting a limited number of high-priority teaching scenarios, and matching each scenario with appropriate teaching and assessment methods. Faculty development should accompany curriculum implementation, particularly when instructors are expected to use simulation, digital technologies, interdisciplinary teaching, and competency-based assessment. Recent evidence indicates that faculty development programs can improve teaching-related knowledge, skills, behaviors, and teaching quality, while continuous feedback is important for sustaining their effects (33). Curriculum evaluation should also extend beyond student satisfaction and specify evaluation objectives, competency outcomes, assessment tools, and standards. A recent review of competency-based medical education found substantial variation and incomplete reporting in curriculum-evaluation objectives, approaches, tools, and standards (34). Future studies should therefore prospectively evaluate aerospace cardiovascular medicine teaching models using outcomes such as knowledge retention, physiological and clinical reasoning, teamwork, aeromedical decision-making, feasibility, resource requirements, and cost. Multicenter and mixed-methods research would be valuable for determining which combinations of teaching strategies are effective and transferable across different institutions.

## 9 Conclusion

Improving the teaching quality of aerospace cardiovascular medicine is necessary to address the growing need for learners who can integrate cardiovascular knowledge with the distinctive physiological, occupational, and operational demands of aviation and spaceflight. This perspective identifies six interrelated priorities: diverse teaching methods, technology-enhanced tools, personalized guidance and support, structured feedback and evaluation, teaching case repositories, and project-based and interdisciplinary education. In practice, institutions can use this framework to define core competencies, develop aerospace cardiovascular scenarios and case resources, match teaching and assessment methods with learning objectives, strengthen faculty development, and establish continuous feedback mechanisms. If appropriately implemented and prospectively evaluated, these measures may improve the consistency, relevance, and transferability of training and better prepare learners for aeromedical assessment, emergency decision-making, and interdisciplinary mission support.

## Data Availability

The original contributions presented in the study are included in the article/supplementary material, further inquiries can be directed to the corresponding author.
